# New Insights into Potential Anti-Aging Effects of a Dietary Supplement from Chlorella Growth Factor and γ-PGA in Aged SAMP8 Mice

**DOI:** 10.3390/biology15060503

**Published:** 2026-03-20

**Authors:** Ming-Yu Chou, Shih-An Yang, Po-Hsien Li, Tzu-Chien Kao, Shih-Yi Wang, Po-Hsun Cheng, Ching-Hsin Chi, Shu-Fen Cheng, Yue-Ching Wong, Ming-Fu Wang

**Affiliations:** 1 International Aging Industry Research & Development Center (AIC), Providence University, Taichung 433303, Taiwan; 2 Product Development & Research Institute, Vedan Biotechnology, Taichung 43351, Taiwan; 3 Department of Food and Nutrition, Providence University, Taichung 433303, Taiwan; 4 Department of Nutrition, Chung Shan Medical University, Taichung 402306, Taiwan

**Keywords:** γ-PGA, chlorella growth factor, SAMP8, anti-aging

## Abstract

Aging is closely associated with oxidative stress, which contributes to functional decline and increased vulnerability to neurodegenerative diseases. Natural antioxidants, such as Chlorella Growth Factor (CGF) and γ-polyglutamic acid (γ-PGA), possess antioxidant and anti-aging properties; however, their combined effects remain unknown. This study investigated the potential synergistic effects of CGF and γ-PGA supplementation in senescence-accelerated mouse-prone 8 (SAMP8) mice, a model characterized by early cognitive decline, locomotor deficits, and elevated oxidative DNA damage. Three-month-old male SAMP8 mice (*n* = 40) were divided into four groups: control, CGF (49.2 mg/kg BW/day), γ-PGA (20.5 mg/kg BW/day), and combined CGF + γ-PGA (69.7 mg/kg BW/day), and were treated for 13 weeks. Behavioral and physiological assessments included locomotor activity, aging index, and cognitive function (passive and active avoidance tests). Biochemical analysis focused on brain 8-hydroxy-2′-deoxyguanosine (8-OHDG) as a biomarker of oxidative DNA damage. Supplementation with CGF and γ-PGA, particularly in combination, significantly improved locomotor activity, aging scores, and cognitive functions. Notably, the combined treatment yielded the greatest reduction in brain 8-OHDG levels. These findings indicate that CGF and γ-PGA, when administered together, exert enhanced protective effects against functional and molecular aging. In conclusion, long-term supplementation with CGF and γ-PGA protects against aging-related decline in SAMP8 mice. This study highlights the potential of CGF and γ-PGA as safe, natural candidates for the development of functional foods or nutraceuticals aimed at promoting healthy aging and reducing oxidative stress-associated disorders.

## Simple Summary

This study aimed to investigate the combined efficacy and potential synergistic benefits of CGF and γ-PGA in SAMP8 mice. Specifically, we assessed behavioral and aging indices, cognitive performance, and brain levels of 8-OHDG to determine whether natural antioxidant supplementation could alleviate both molecular damage and functional impairment associated with aging. This integrative approach provides new insights into the role of combined nutritional strategies in promoting healthy aging.

## 1. Introduction

Natural antioxidants have been extensively investigated for their potential to mitigate oxidative stress, a central driver of aging and age-related diseases. Chlorella growth factor (CGF), a water-soluble extract of *Chlorella sorokiniana*, contains nucleic acids, peptides, vitamins, and chlorophyll derivatives [1] and has been reported to promote tissue repair, enhance immune responses [2], and exert antioxidant effects [3]. Similarly, γ-polyglutamic acid (γ-PGA), a biopolymer produced by *Bacillus* species [4], has demonstrated antioxidant, immunomodulatory, and metabolic regulatory properties [5,6]. While both CGF and γ-PGA individually exhibit anti-aging properties [7,8], studies examining their combined effects on aging are limited.

Globally, population aging has emerged as a pressing challenge, with the proportion of individuals aged 65 years and older rapidly increasing [9]. This demographic shift is accompanied by a higher prevalence of age-related disorders, including cognitive decline, frailty, and metabolic dysfunction, all of which place a significant burden on healthcare systems [10]. Consequently, there is a growing interest in identifying safe and effective strategies to slow functional deterioration associated with aging. The senescence-accelerated mouse prone 8 (SAMP8) strain is a valuable preclinical model. SAMP8 mice exhibit early-onset cognitive decline, reduced locomotor activity, and visible signs of senescence, along with heightened oxidative stress and accumulation of oxidative DNA damage, such as 8-hydroxy-2′-deoxyguanosine (8-OHDG) [11]. This combination of behavioral deficits and molecular alterations makes the SAMP8 model highly relevant for studying interventions targeting age-related decline.

Given the increasing need for interventions that link biochemical protection with functional outcomes, this study aimed to investigate the combined efficacy and potential synergistic benefits of CGF and γ-PGA in SAMP8 mice. Specifically, we assessed behavioral and aging indices, cognitive performance, and brain levels of 8-OHDG to determine whether natural antioxidant supplementation could alleviate both molecular damage and functional impairment associated with aging. This integrative approach provides new insights into the role of combined nutritional strategies in promoting healthy aging.

## 2. Material and Methods

### 2.1. Sample Preparation

CGF was prepared from *C. sorokiniana* VB-1974 biomass following the method described by [12]. Briefly, the biomass was extracted in boiling water (100 °C) for 20 min and centrifuged (8000× *g*, 30 min) using a centrifuge (SCR 20B, Hitachi, Ltd., Tokyo, Japan). The supernatant was filtered to obtain the CGF extract. γ-PGA was produced by *Bacillus subtilis* 91CT227, as described by [13]. Following centrifugation (15,100× *g*, 15 min), the supernatant was treated with ethanol overnight at 4 °C to precipitate γ-PGA. The precipitate was then redissolved, dialyzed (MWCO 8000–14,000 Da) (Merck KGaA, Darmstadt, Germany), and lyophilized (Glass Vials Australia PTY Ltd., Wetherill Park, Australia). The CGF from *C. sorokiniana* VB-1974 and γ-PGA from *B. subtilis* 91CT227 were provided by Vedan Biotechnology (Taichung, Taiwan).

### 2.2. Animal and Experiment Design

Three-month-old male SAMP8 mice were used in this study. A total of 40 animals were randomly assigned to four groups (*n* = 10 per group): (1) control group, administered distilled water twice daily via oral gavage; (2) the CGF group, receiving 49.2 mg/kg BW/day of CGF; (3) the γ-PGA group, receiving 20.5 mg/kg BW/day of γ-PGA; and (4) combined group, receiving 69.7 mg/kg BW/day of CGF and γ-PGA. The dosages administered in this study were determined based on the recommended daily intake for humans, following the guidelines set forth in the ‘Health Food Evaluation Methods for Anti-aging Efficacy’ by the Ministry of Health and Welfare, Taiwan. A conversion factor of 12.3 (based on body surface area normalization for a 60 kg adult) was applied to calculate the murine-equivalent dose. All treatments were administered via gavage twice daily throughout the 13-week experimental period. At Week 11, locomotor activity and aging index assessments were conducted. In Week 12, a single-trial passive avoidance test was performed to evaluate memory retention. During Week 13, an active avoidance test was conducted, followed by the sacrifice of the animals for the collection of brain tissues to assess the levels of 8-OHDG, a marker of oxidative DNA damage.

All SAMP8 mice were housed in a 30 (W) × 20 (D) × 10 (H) cm^3^ transparent plastic cage placed in an automatically controlled, dust-free room at a temperature of 22 ± 2 °C and relative humidity of 65 ± 5%. An automatic timer managed the light cycle from 07:00 a.m. to 7:00 p.m. under light and from 7:00 p.m. to 7:00 a.m. in the dark. The animals were fed ad libitum with AIN-93M standard purified feed and water, which were changed every other day [14]. The experimental animal protocol was approved by the Animal Research Committee of the University of Providence under IACUC Approval No. 20170512-A01, and the approval date was 12 May 2017. All operation procedures followed the standards outlined in the Committee for the Control and Supervision of Animal Experiments and the National Institutes of Health Guide for the Care and Use of Laboratory Animals.

### 2.3. Open Field Activity Test

A locomotor activity test was conducted during the 11th week of the experiment, prior to initiating cognitive behavioral assessments. Each mouse was individually placed at the center of an aluminum activity chamber (25 cm W × 25 cm D × 25 cm H; Activity Monitor Video Path Analyzer, Coulbourn Instruments, Whitehall Township, PA, USA, Model E61-21), and its spontaneous horizontal movement was monitored. The test was performed under controlled conditions in a dimly lit, acoustically isolated room to minimize external stimuli. Locomotor activity was continuously recorded using an automated video tracking system. The total duration of horizontal movement (locomotion; seconds per 5 min interval) was quantified, with data collected at 5 min intervals over a 10 min testing period for each animal.

### 2.4. Assessment of Aging Index

An aging index assessment was performed on the 11th week of the experiment, following the criteria established by a previous study [15]. The evaluation included four domains: behavior (exploratory reactivity within 30 s and response to dorsal skin pinching), appearance (hair glossiness, coarseness, alopecia, and presence of skin ulcers), ocular condition (periophthalmic inflammation or eyelid edema), and spinal curvature (lordosis or kyphosis assessed by inspection and palpation). Each parameter was scored on a five-point scale ranging from 0 to 4, with higher scores indicating more severe age-related changes. The total aging score was calculated by summing all individual item scores to provide an overall index of aging severity.

### 2.5. Single-Trial Passive Avoidance Test

In the 12th week of the experiment, a single-trial passive avoidance test was performed to evaluate the learning and memory functions. The apparatus consisted of a shuttle cage (35 cm W × 17 cm D × 20 cm H; Coulbourn Instruments, Model E10-15) divided into a brightly lit chamber and a dark chamber separated by a guillotine door (7.5 cm W × 6.5 cm D; Model E10-15GD). The floor of the apparatus was composed of parallel stainless-steel rods (1 cm apart) connected to a shock generator.

During the training phase, each mouse was placed in an illuminated chamber for a 10 s acclimation period. The guillotine door was then opened to allow free exploration of the two chambers by the rats. Owing to their innate preference for dark environments, mice typically enter the dark chamber. Upon full entry, the door was immediately closed, and after 5 s, the animal received a mild foot shock (0.5 mA for 0.5 s), which was repeated three times at 5 s intervals. The latency to enter the dark chamber was recorded as a measure of training.

Memory retention was assessed at 24, 48, and 72 h post-training using the same procedure, but without delivering any foot shock. A maximum cutoff time of 180 s was used. A longer latency to enter the dark chamber during retention trials was interpreted as better memory performance.

### 2.6. Active Shuttle Avoidance Test

In the 13th week of the experiment, an active avoidance test was performed to assess cognitive flexibility and associative learning. The test was performed using a shuttle cage measuring 35 cm in width, 17 cm in depth, and 20 cm in height (Coulbourn Instruments, Model E10 15). The apparatus consisted of two chambers connected by a small guillotine door. The floor was composed of stainless-steel rods spaced one centimeter apart and connected to a current generator. The entire procedure, including the timing, presentation of visual and auditory cues, and delivery of shocks, was controlled using a computer-based system.

During the test, each mouse was placed in one chamber and allowed a 10 s adaptation period, referred to as the intertrial interval. A conditioned stimulus consisting of light and sound was then presented for 10 s. If the mouse remained in the original chamber without responding during the conditioned stimulus, it received a single unconditioned stimulus consisting of a 0.3 milliampere foot shock that lasted 5 s. If the mouse moved to the opposite chamber during the conditioned stimulus, a shock was not administered.

### 2.7. Determination of 8-Hydroxydeoxyguanosine (8-OHDG) Levels

To determine 8-OHDG levels, 30 mg of brain tissue was used with a genomic DNA isolation kit (SN026-0100, GeneDireX, Inc., Miaoli County, Taiwan). The level of 8-OHDG in the samples was determined using an 8-OHDG ELISA kit (8-OHDG, ab285254, Abcam, Waltham, MA, USA) according to the manufacturer’s instructions.

### 2.8. Statistical Analysis

All values in this study are expressed as mean ± standard error of the mean (S.E.M.). Data were analyzed using SAS (ver. 9.4, SAS Institute Inc., Cary, NC, USA). Prior to analysis, the data were tested for normality and homogeneity of variance. One-way analysis of variance (ANOVA) was used to test the differences between groups. Duncan’s multiple range test was used to assess significant differences between groups (*p* < 0.05).

## 3. Results

### 3.1. General Physiological and Organ Parameters

After 13 weeks of treatment, no significant differences were observed among the groups in terms of final body weight, food intake, or water consumption (Table 1). All mice exhibited stable growth during the experimental period. Group B (CGF) exhibited the greatest body weight gain (1.93 ± 0.70 g), whereas Group C (γ-PGA) showed the lowest (1.04 ± 0.40 g). Food intake ranged from 5.52 to 5.73 g/day, and water consumption remained between 6.47 and 6.61 mL/day, indicating that neither CGF nor γ-PGA affected feeding behavior or hydration status.

Organ weights, expressed as g per 100 g of body weight, were also unaffected by the treatments (Table 2). No significant differences were observed in the brain, heart, liver, spleen, lung, or kidney weights between the groups. Slight variations included marginally lower liver weights in Groups B and D (both 4.81 g/100 g BW) and a slightly higher spleen weight in Group C (0.36 ± 0.05 g), but these were not statistically significant. These findings suggest that the administration of CGF and γ-PGA, individually or in combination, did not adversely affect general physiological growth or organ development.

### 3.2. Behavioral and Aging Assessments

Spontaneous locomotor activity was assessed during week 11 to evaluate baseline behavioral responsiveness. As shown in Table 3, all treatment groups exhibited higher locomotor activity during the first 5 min than the control group. Group C (γ-PGA) demonstrated the highest activity (108.40 ± 6.17 s), followed closely by Group D (CGF + γ-PGA, 106.00 ± 2.65 s). A similar trend was observed in the 6–10 min interval, suggesting that supplementation with CGF and γ-PGA may help sustain exploratory behavior and reduce age-related declines in physical activity.

In the same week, aging phenotypes were evaluated using a standardized aging index scoring system based on behavioral, physical, ocular, and spinal characteristics (Table 4). The total aging index score was significantly reduced in all treatment groups compared to that in the control group, with Group D showing the lowest score (4.70 ± 0.40 vs. 7.20 ± 0.39 in the control group, *p* < 0.05). Improvements were especially notable in hair loss, periophthalmic conditions, and spinal curvature. These findings indicate that CGF and γ-PGA, particularly in combination, attenuate both functional and morphological signs of aging in SAMP8 mice.

### 3.3. Cognitive Performance Assessments

**Figure 1:**
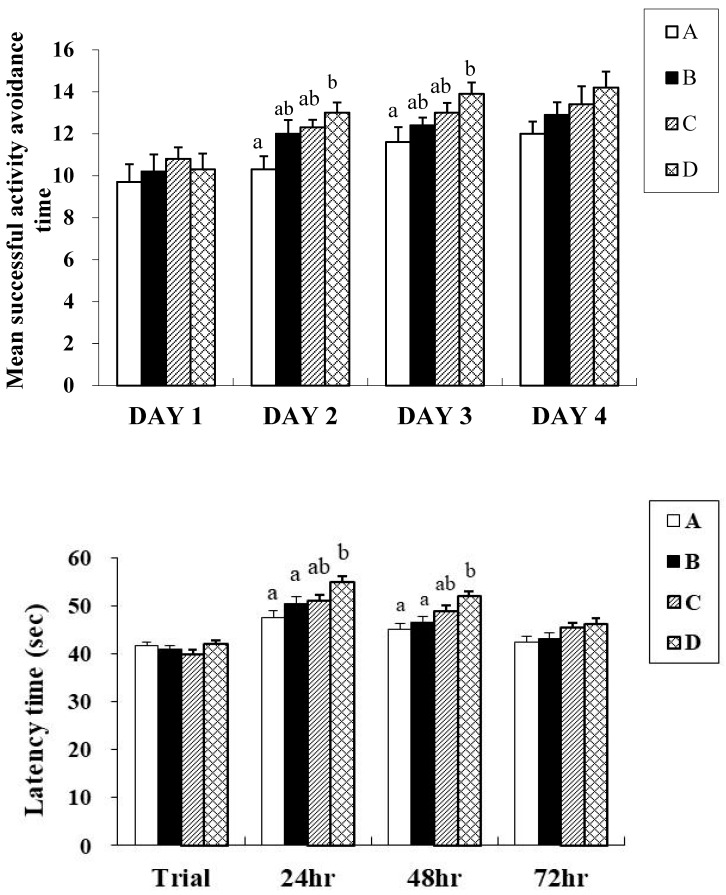
Active avoidance and latency time of 3-month-old male SAMP8 mice after treatment with CGF and γ-PGA powder. Values were expressed as mean ± S.E.M. and analyzed by one-way ANOVA (*n* = 10). Means within a column followed by different superscript letters differ significantly (*p* < 0.05) according to Duncan’s multiple range test. A = Control (double-distilled water). B = 49.2 mg/kg BW/day of CGF powder. C = 20.5 mg/kg BW/day of γ-PGA powder. D = 69.7 mg/kg BW/day of CGF + γ-PGA powder.

Cognitive function was evaluated using passive and active avoidance paradigms in the study. In the passive avoidance test (Table 5; Figure 1), latency to enter the dark chamber was used as an indicator of memory retention. At 24, 48, and 72 h after training, all treatment groups demonstrated longer latencies than the control group. The combination group (Group D) exhibited the most pronounced improvement, with a latency of 46.20 ± 1.25 s at 72 h, which was significantly longer than that of the control group (42.30 ± 1.32 s, *p* < 0.05). These findings suggest that CGF and γ-PGA supplementation enhances the retention of aversive memory, with the combined treatment showing the greatest effect.

In the active avoidance test (Table 6; Figure 1), the number of successful avoidances increased progressively over the four testing days. Group D again outperformed the other groups, reaching 14.20 ± 0.76 successful avoidances on day 4, which was significantly higher than that of the control group (12.00 ± 0.58, *p* < 0.05). Groups B and C also showed improvements, although to a lesser extent. These results further support the cognitive-enhancing effects of CGF and γ-PGA, particularly in the context of associative learning and memory retention under repeated stimulation.

### 3.4. Oxidative DNA Damage

**Figure 2:**
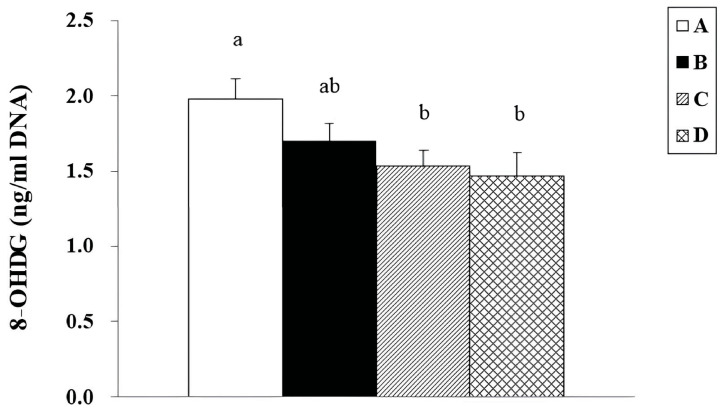
Levels of 8-OHDG in the brain of 3-month-old male SAMP8 mice after 13 weeks of treatment with CGF and γ-PGA powder. Values were expressed as mean ± S.E.M. and analyzed by one-way ANOVA. (*n* = 10). Means within a column followed by different superscript letters differ significantly (*p* < 0.05) according to Duncan’s multiple range test. A = Control (double-distilled water). B = 49.2 mg/kg BW/day of CGF powder. C = 20.5 mg/kg BW/day of γ-PGA powder. D = 69.7 mg/kg BW/day of CGF + γ-PGA powder.

Oxidative DNA damage in the brain was evaluated by measuring the levels of 8-OHdG, a widely recognized biomarker of oxidative stress. As shown in Table 7 and Figure 2, 8-OHDG levels were significantly lower in Groups C and D than in the control group. Group D (CGF + γ-PGA) exhibited the lowest level (1.46 ± 0.16 ng/mg protein), followed by Group C (1.53 ± 0.11 ng/mg), both significantly reduced compared to Group A (1.98 ± 0.14 ng/mg, *p* < 0.05). These findings indicate that CGF and γ-PGA, especially when combined, effectively suppress oxidative DNA damage in the brains of aging SAMP8 mice.

## 4. Discussions

### 4.1. Antioxidant Supplementation and Delayed Aging Phenotypes

The improvement in locomotor activity and reduction in aging index scores in CGF and γ-PGA-treated SAMP8 mice suggest that antioxidant supplementation may attenuate the functional signs of aging. Unlike many antioxidant studies that focus solely on molecular endpoints, this approach links systemic redox modulation to the observable phenotypes. Previous reports using the SAMP8 model have emphasized its utility in evaluating the visible and behavioral markers of senescence, including hair loss and spinal curvature [16]. Our results extend this evidence by demonstrating that natural antioxidants can influence these parameters. This implies that oxidative stress contributes not only to molecular deterioration but also to phenotypic aging of the skin. Nevertheless, the precise mechanisms by which redox balance translates into improvements in signs of external aging remain unclear. For instance, the effects of antioxidant supplementation on dermal fibroblast function and skeletal muscle oxidative load have not been well studied, representing a gap in future investigations.

### 4.2. Cognitive Enhancement Through Synergistic Antioxidant Effects

The enhanced memory retention observed in the passive avoidance test and improved associative learning in the active avoidance test highlight the cognitive benefits of CGF and γ-PGA. Previous studies have demonstrated that *Chlorella sorokiniana* supplementation can ameliorate cognitive decline in aging and neurodegenerative models, likely by reducing oxidative stress and promoting neurotrophic support [17,18]. Similarly, γ-PGA has been reported to exert indirect neuroprotective effects through gut microbiota modulation and systemic antioxidant enhancement [19]. Importantly, our findings showed that the combination of CGF and γ-PGA resulted in the most consistent improvement. These results are consistent with previous studies on combined antioxidant interventions. For instance, Chou et al. (2022) [20] reported that a mixed antioxidant supplement significantly improved cognitive function and coordination in aged mice by simultaneously targeting multiple physiological pathways, yielding superior outcomes compared with single-nutrient interventions. Similarly, classical studies on antioxidant combinations, such as Vitamin E and C, have demonstrated that multi-target strategies can provide additive protection against oxidative stress-induced cognitive decline [21,22]. This enhanced efficacy suggests a potential synergistic effect arising from the complementary mechanisms. Specifically, CGF directly provides nucleic acids and peptides to support cellular repair and reduce oxidative damage, whereas γ-PGA likely exerts neuroprotection indirectly via the gut–brain axis and systemic immune modulation. This provides evidence that targeting multiple pathways simultaneously through both direct cellular defense and systemic regulation may yield stronger cognitive protection than that provided by single agents. However, as our study did not include hippocampal-specific assays or synaptic protein analysis, the neurobiological substrates of these behavioral improvements remain unknown.

### 4.3. Oxidative DNA Damage as a Mechanistic Basis for Functional Preservation

The significant reduction in 8-OHDG levels in the brain provides molecular evidence that complements these behavioral outcomes. 8-OHDG is a widely recognized biomarker of oxidative DNA damage and is strongly associated with cognitive decline and neurodegeneration [23]. Our findings are consistent with those of studies reporting that antioxidant supplementation reduces oxidative DNA lesions and delays cognitive impairment in aged models [24]. Importantly, the parallel improvement in memory performance and reduction in 8-OHDG levels suggest that alleviating DNA damage may be a key mechanism underlying functional preservation. However, the specific contributions of DNA repair pathways and ROS reduction are unclear. For example, the activation of the Nrf2 pathway has been implicated in antioxidant-mediated protection of DNA integrity [25]; however, this was not evaluated in the present study. Future studies incorporating DNA repair enzyme activity and mitochondrial redox assays will provide clearer mechanistic links.

Furthermore, the mechanistic basis of these anti-aging effects may involve the mammalian target of the rapamycin (mTOR) signaling pathway. The mTOR pathway plays a critical role in regulating aging by integrating nutrient sensing, oxidative stress responses, and autophagy. Chronic or dysregulated mTOR activation accelerates aging phenotypes by suppressing autophagy and the accumulation of oxidative DNA damage [26,27]. In the present study, the significant reduction in brain 8-OHDG levels and improved cognitive performance following combined CGF and γ-PGA supplementation suggest that these beneficial effects may be mediated through the modulation of mTOR-related pathways. This hypothesis is supported by recent studies indicating that specific nutritional interventions can attenuate age-related cognitive decline and oxidative stress by regulating mTOR signaling and restoring the autophagic flux [27,28]. Therefore, the neuroprotective effects observed in this study likely involve the coordinated regulation of oxidative defense systems and central aging signaling networks.

## 5. Conclusions

This study showed that CGF and γ-PGA (provided by Vedan Biotechnology), especially when administered in combination, alleviated aging-associated functional decline in SAMP8 mice. Improvements in locomotor activity, reductions in aging index scores, enhanced performance in memory-related behavioral tasks, and decreased oxidative DNA damage were accompanied by the activation of endogenous antioxidant defense systems. These outcomes highlight the importance of targeting oxidative stress as a central mechanism of age-related deterioration and demonstrate the value of combining CGF and γ-PGA to achieve synergistic effects.

Compared with prior studies reporting modest benefits of CGF or γ-PGA alone, our results extend the literature by demonstrating that their combination, which called Chlorich^®^ AgeDefy, provides more consistent improvements across the behavioral, physiological, and biochemical levels. Given their natural origin and favorable safety profile, the use of CGF and γ-PGA in multi-component nutritional interventions may offer greater efficacy than single-agent strategies and holds practical potential for development into functional foods, nutraceuticals, or dietary supplements aimed at promoting healthy aging and reducing the burden of age-related diseases.

## Figures and Tables

**Table 1:** Body weight, food intake and water consumption of 3-month-old male SAMP8 mice after 13 weeks of treatment with CGF and γ-PGA powder ^1,2^.

Group	Body Weight (g)			Food Intake (g/day)	Water Consumption (mL/day)
	Initial	Final	Gain		
A	29.25 ± 0.53	30.91 ± 0.49	1.66 ± 0.32	5.73 ± 0.11	6.61 ± 0.08
B	29.35 ± 1.16	31.28 ± 0.70	1.93 ± 0.70	5.66 ± 0.06	6.47 ± 0.09
C	29.23 ± 0.46	30.27 ± 0.31	1.04 ± 0.40	5.52 ± 0.08	6.54 ± 0.06
D	29.49 ± 0.97	30.78 ± 0.91	1.28 ± 0.30	5.57 ± 0.07	6.51 ± 0.07

^1^ Values were expressed as mean ± S.E.M. and analyzed by one-way ANOVA (*n* = 10). ^2^ No significant differences were observed among the groups (*p* > 0.05). A = Control (double-distilled water). B = 49.2 mg/kg BW/day of CGF powder. C = 20.5 mg/kg BW/day of γ-PGA powder. D = 69.7 mg/kg BW/day of CGF + γ-PGA powder.

**Table 2:** The organ weight of 3-month-old male SAMP8 mice after 13 weeks of treatment with CGF and γ-PGA powder ^1,2^.

Sex	Group (*n* = 10)	Organ Weight (g/100 g Body Weight)					
		Brain	Heart	Liver	Spleen	Lung	Kidney
Male	A	1.58 ± 0.03	0.61 ± 0.01	5.04 ± 0.10	0.31 ± 0.01	0.66 ± 0.04	1.68 ± 0.03
	B	1.59 ± 0.04	0.60 ± 0.02	4.81 ± 0.17	0.30 ± 0.03	0.67 ± 0.03	1.64 ± 0.04
	C	1.54 ± 0.02	0.57 ± 0.03	5.08 ± 0.37	0.36 ± 0.05	0.63 ± 0.03	1.64 ± 0.02
	D	1.50 ± 0.03	0.59 ± 0.03	4.81 ± 0.41	0.33 ± 0.04	0.68 ± 0.02	1.62 ± 0.06

^1^ Values were expressed as mean ± S.E.M. and analyzed by one-way ANOVA (*n* = 10). ^2^ No significant differences were observed among the groups (*p* > 0.05). A = Control (double-distilled water). B = 49.2 mg/kg BW/day of CGF powder. C = 20.5 mg/kg BW/day of γ-PGA powder. D = 69.7 mg/kg BW/day of CGF + γ-PGA powder.

**Table 3:** The change in locomotion of 3-month-old male SAMP8 mice after 11 weeks of treatment with CGF and γ-PGA powder ^1,2^.

Group	Locomotion	
	Time Interval [6]	
	0–5	6–10
	(s/5 min)	
A	96.10 ± 5.55	77.20 ± 4.47
B	101.90 ± 3.75	83.60 ± 2.86
C	108.40 ± 6.17	83.60 ± 4.43
D	106.00 ± 2.65	79.30 ± 2.90

^1^ Values were expressed as mean ± S.E.M. and analyzed by one-way ANOVA (*n* = 10). ^2^ No significant differences were observed among the groups (*p* > 0.05). A = Control (double-distilled water). B = 49.2 mg/kg BW/day of CGF powder. C = 20.5 mg/kg BW/day of γ-PGA powder. D = 69.7 mg/kg BW/day of CGF + γ-PGA powder.

**Table 4:** Aging index of 3-month-old male SAMP8 mice after 11 weeks of treatment with CGF and γ-PGA powder ^1,2^.

Group	A	B	C	D
Behavior				
Reactivity	1.10 ± 0.23	0.90 ± 0.18	0.80 ± 0.13	0.70 ± 0.21
Passivity	0.70 ± 0.21	0.60 ± 0.22	0.60 ± 0.22	0.50 ± 0.17
Skin				
Glossiness	1.00 ± 0.26	0.90 ± 0.18	0.80 ± 0.13	0.80 ± 0.20
Coarseness	0.90 ± 0.18	0.90 ± 0.18	0.80 ± 0.13	0.80 ± 0.13
Hair loss	1.10 ± 0.10 ^a^	0.50 ± 0.17 ^b^	0.50 ± 0.17 ^b^	0.50 ± 0.22 ^b^
Ulcer	0.50 ± 0.22	0.50 ± 0.22	0.30 ± 0.15	0.30 ± 0.15
Eyes				
Periophthalmic lesion	0.80 ± 0.25	0.70 ± 0.15	0.50 ± 0.22	0.40 ± 0.16
Spine				
Lordokyphosis	1.10 ± 0.18	1.00 ± 0.15	0.80 ± 0.25	0.70 ± 0.15
Total	7.20 ± 0.39 ^a^	6.00 ± 0.58 ^ab^	5.10 ± 0.35 ^b^	4.70 ± 0.40 ^b^

^1^ Values were expressed as mean ± S.E.M. and analyzed by one-way ANOVA (*n* = 10). ^2^ Means within a row followed by different superscript letters differ significantly (*p* < 0.05) according to Duncan’s multiple range test. A = Control (double-distilled water). B = 49.2 mg/kg BW/day of CGF powder. C = 20.5 mg/kg BW/day of γ-PGA powder. D = 69.7 mg/kg BW/day of CGF + γ-PGA powder.

**Table 5:** The change in latency time of 3-month-old male SAMP8 mice after 12 weeks of treatment with CGF and γ-PGA powder ^1,2^.

Group	Latency Time (s)			
	Trial	24 h	48 h	72 h
A	41.60 ± 0.90	47.60 ± 1.30 ^a^	45.00 ± 1.26 ^a^	42.30 ± 1.32
B	40.80 ± 0.85	50.50 ± 1.38 ^a^	46.50 ± 1.20 ^a^	43.20 ± 1.21
C	39.90 ± 0.96	51.10 ± 1.32 ^ab^	48.70 ± 1.37 ^ab^	45.30 ± 1.27
D	41.90 ± 0.90	54.90 ± 1.32 ^b^	51.90 ± 1.25 ^b^	46.20 ± 1.25

^1^ Values were expressed as mean ± S.E.M. and analyzed by one-way ANOVA (*n* = 10). ^2^ Means within a column followed by different superscript letters differ significantly (*p* < 0.05) according to Duncan’s multiple range test. A = Control (double-distilled water). B = 49.2 mg/kg BW/day of CGF powder. C = 20.5 mg/kg BW/day of γ-PGA powder. D = 69.7 mg/kg BW/day of CGF + γ-PGA powder.

**Table 6:** Active avoidance of 3-month-old male SAMP8 mice after 13 weeks of treatment with CGF and γ-PGA powder ^1,2^.

Group	Mean Successful Activity Avoidance Time			
	Day 1	Day 2	Day 3	Day 4
A	9.70 ± 0.84	10.30 ± 0.63 ^a^	11.60 ± 0.72 ^a^	12.00 ± 0.58
B	10.20 ± 0.81	12.00 ± 0.65 ^ab^	12.40 ± 0.37 ^ab^	12.90 ± 0.60
C	10.80 ± 0.55	12.30 ± 0.37 ^ab^	13.00 ± 0.47 ^ab^	13.40 ± 0.86
D	10.30 ± 0.76	13.00 ± 0.49 ^b^	13.90 ± 0.55 ^b^	14.20 ± 0.76

^1^ Values were expressed as mean ± S.E.M. and analyzed by one-way ANOVA (*n* = 10). ^2^ Means within a column followed by different superscript letters differ significantly (*p* < 0.05) according to Duncan’s multiple range test. A = Control (double-distilled water). B = 49.2 mg/kg BW/day of CGF powder. C = 20.5 mg/kg BW/day of γ-PGA powder. D = 69.7 mg/kg BW/day of CGF + γ-PGA powder.

**Table 7:** Levels of 8-OHDG in the brain of 3-month-old male SAMP8 mice after 13 weeks of treatment with CGF and γ-PGA powder ^1,2^.

Group (*n* = 10)	8-OHDG
	Male
A	1.98 ± 0.14 ^a^
B	1.70 ± 0.12 ^ab^
C	1.53 ± 0.11 ^b^
D	1.46 ± 0.16 ^b^

^1^ Values were expressed as mean ± S.E.M. and analyzed by one-way ANOVA (*n* = 10). ^2^ Means within a column followed by different superscript letters differ significantly (*p* < 0.05) according to Duncan’s multiple range test. A = Control (double-distilled water). B = 49.2 mg/kg BW/day of CGF powder. C = 20.5 mg/kg BW/day of γ-PGA powder. D = 69.7 mg/kg BW/day of CGF + γ-PGA powder.

## Data Availability

The data presented in this study are available on request from the corresponding author. The data are not publicly available due to privacy and ethical restrictions.
